# Association of obesity with the development of end stage renal disease in IgA nephropathy patients

**DOI:** 10.3389/fendo.2023.1094534

**Published:** 2023-03-20

**Authors:** Siqing Wang, Aiya Qin, Lingqiu Dong, Jiaxing Tan, Xiaoyuan Zhou, Wei Qin

**Affiliations:** ^1^ West China School of Medicine, Sichuan University, Chengdu, Sichuan, China; ^2^ Division of Nephrology, Department of Medicine, West China Hospital of Sichuan University, Chengdu, Sichuan, China; ^3^ West China School of Public Health and West China fourth hospital, Sichuan University, Chengdu, Sichuan, China

**Keywords:** IgA nephropathy, obesity, end stage renal disease, renal outcome, renal prognosis

## Abstract

**Background and aim:**

Immunoglobulin A nephropathy (IgAN) is the most common primary glomerulonephritis worldwide. We aimed to evaluate whether obesity is a risk factor for IgAN patients.

**Methods:**

A total of 1054 biopsy-proven IgAN patients were analyzed in this retrospective study. Patients were divided into four groups according to their body weight index (BMI) at the period of renal biopsy: underweight group (BMI< 18.5, N=75), normal weight group (18.5≤BMI<24, N=587), overweight group (24≤BMI<28, N=291) and obesity group (28≤BMI, N=101). The endpoint of our study was end stage renal disease (ESRD: eGFR <15 mL/min/1.73 m^2^ or having renal replacement treatment). Kaplan-Meier analyses and Cox proportional hazard models were performed to evaluate renal survival. Propensity-score matching (PSM) was performed to get the matched cohort to evaluate the role of obesity in IgAN patients. Besides, the effect modification of obesity and hypertension in IgAN patients was clarified by the synergy index.

**Results:**

IgAN patients complicated with obesity had more severe renal dysfunction at the time of renal biopsy than those with optimal body weight. In addition, patients with obesity tended to have higher risk of metabolic disorders, such as hyperuricemia (64.4% vs 37%, p<0.001), hypertriglyceridemia (71.3% vs 32.5%, p<0.001) and hypercholesterolemia (46.5% vs 35.6%, p=0.036). It was observed that obesity patients had higher rate of unhealthy behaviors, such as smoking (27.7% vs 16.4%, p=0.006) and alcohol drinking (29.7% vs 19.9%, p=0.027). Although obesity was not confirmed as an independent risk factor for IgAN patients, we found that IgAN patients with obesity presented with higher incidence of hypertension, as well as lower event-free renal survival rate (log-rank p < 0.001), especially in patients with 24-h urine protein ≥ 1g (log-rank p =0.002). In addition, the synergy index showed that there was positive interaction between obesity and hypertension in IgAN.

**Conclusion:**

Obesity is an important risk factor for IgAN patients when combined with hypertension. Hypertension appears to be common in obese IgAN patients.

## Introduction

The prevalence of overweight and obesity have increasing impact on the quality of life of patients, on health services, and on society (1). Recent studies have suggested that elevated body mass index (BMI) was a significant risk factor for coronary heart disease, stroke (2) and chronic kidney disease (CKD) (3). Immunoglobulin A nephropathy (IgAN) is the most common primary glomerulonephritis globally and represents the leading cause of CKD and renal failure (4). The relationship between obesity and IgAN has not yet been clarified clearly because controversial results have been obtained in previous studies. Several studies have showed that overweight and obesity might be related to severe renal dysfunction and poor prognosis (5–7). However, other studies suggested that high BMI was not a direct risk factor for IgAN (8, 9). Therefore, we performed this study to determine whether obesity plays a role in the progression and prognosis of IgAN in Chinese patients.

## Materials and methods

### Study design

**Figure 1:**
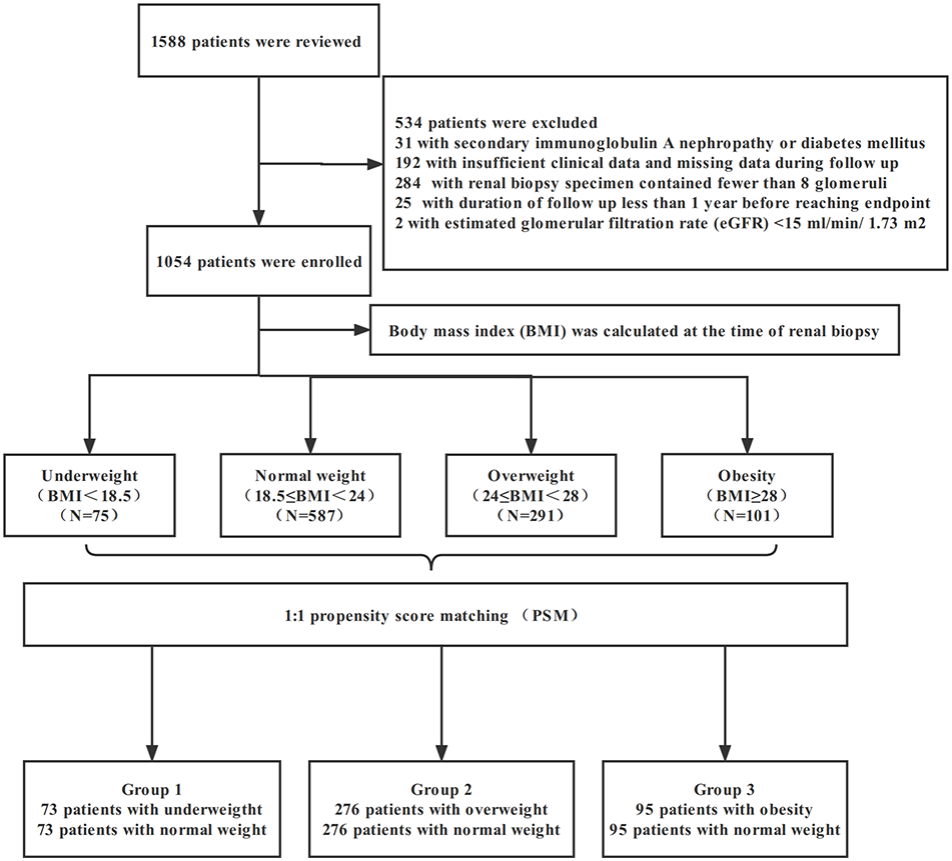
The flow chart of excluded patients.

A total of 1588 renal biopsy proven adult IgAN patients from West China Hospital of Sichuan University between January 2009 and December 2018 were recruited for this retrospective study. Patients with systemic lupus erythematosus, Henoch–Schonlein purpura, chronic liver disease and systemic diseases (including diabetes mellitus) were excluded (31 patients). The other exclusion criteria were as follows (1): insufficient clinical data and missing data during follow up (192 patients) (2), renal biopsy specimens containing <8 glomeruli (284 patients) (3), duration of follow-up <1 year before reaching end stage renal disease (ESRD: eGFR <15 mL/min/1.73 m^2^ or having renal replacement treatment) (25 patients), and (4) estimated glomerular filtration rate (eGFR) <15 ml/min/1.73 m^2^ at the time of renal biopsy (2 patients). Ultimately, 1054 patients were included (**Figure 1**). Written informed consent was obtained from each patient involved. This study was approved by the Ethical Committee of West China Hospital of Sichuan University (2019–33).

### Clinical data

Patients enrolled in this study were divided into four groups according to their BMI at the period of renal biopsy. BMI is calculated as weight in kilograms divided by the measured height in square meters and expressed in kg/m^2^. It remains a good parameter to appreciate excessive weight and the classification is as follows based on WHO (World Health Organization) classification: underweight group (BMI < 18.5), normal weight group (18.5 ≤ BMI < 24), overweight group (24 ≤ BMI < 28), and obesity group (BMI ≥ 28) (10). Demographics (gender, age), clinical data [systolic blood pressure (SBP), diastolic blood pressure (DBP), serum creatinine (Scr), hemoglobin (Hb), serum albumin (Alb), smoking and drinking status, serum total cholesterol (TC), serum triglycerides (TG), uric acid (UA), 24-h urine protein and eGFR] were collected at the time of renal biopsy. Hypertension was defined as blood pressure > 140/90 mmHg or using antihypertensive agents. Mean arterial pressure (MAP) was calculated as DBP + 1/3(SBP-DBP) (11). eGFR was calculated using the CKD-EPI equation (12). Anemia was defined as Hb <120 g/L in males or Hb <110 g/L in females (13). Hyperuricemia was defined as UA >420 µmol/L or >360 µmol/L in males and females (14). Hypercholesterolemia was defined as serum TC ≥ 5.2 mmol/L. Hypertriglyceridemia was defined as serum TG ≥1.7 mmol/L (14). Stages of CKD were based on the Kidney Disease: Improving Global Outcomes (KDIGO) practice guidelines (15).

### Pathological data

Renal biopsy samples were evaluated by light (HE, PAS, Masson, PASM), immunofluorescence (IgA, IgG, IgM, C3, C4, C1q) and electron microscopy. All the renal pathology reports were evaluated by our hospital experienced pathologists and nephrologists according to the Oxford classification of IgAN (MEST-C): mesangial hypercellularity (M0/M1); endocapillary hypercellularity (E0/E1); segmental glomerulosclerosis (S0/S1); tubular atrophy/interstitial fibrosis (T0/T1/T2) and cellular or fibrocellular crescents (C0/C1/C2) (16). We also performed clinical-pathological discussion for all patients, especially for those with difficulty of diagnosis.

### Treatment data

According to different treatment strategies, patients were divided into two groups: supportive treatment, corticosteroids alone or/and plus immunosuppressant therapy. Patients in the supportive treatment group only received optimized supportive care with a full dose angiotensin-converting-enzyme inhibitor (ACEI) or angiotensin receptor blockers (ARB) to achieve target blood pressure. Patients in the corticosteroids alone or/and plus immunosuppressant therapy group received optimized supportive care along with corticosteroids (0.5 - 1 mg/kg prednisone daily, tapering down within 6 - 8 months) or/and immunosuppressant therapy (cyclophosphamide 2 mg/kg daily for 3 months, or mycophenolate mofetil 1-2 g daily for 6-8 months, or cyclosporine 3–5 mg/kg daily for 6–8 months, or tacrolimus 0.03–0.05 mg/kg daily for 6–8 months) (17).

### Endpoint

The main predefined study outcome for the present analysis was the occurrence of end stage renal disease (ESRD), which was defined as eGFR <15 mL/min/1.73 m2 or accepting renal replacement treatment.

### Statistical analysis

All the statistical analyses were carried out by using IBM SPSS Statistic software (version 26.0). Continuous variables were expressed as the means ± standard deviations (SD) or median with interquartile range and analyzed with independent samples t-test or nonparametric test for normally and nonnormally distributed variables. Categorical data were analyzed using Chi-square tests and presented as frequencies (percentages). Kaplan-Meier analyses and Cox proportional hazard models were performed to evaluate the risk factors for renal progression and prognosis, and survival curves were compared with the log-rank test. Combining the results of the univariate Cox proportional hazard model and clinical experience, we screened out confounding variables and added them to the different multivariate Cox proportional hazard models. In the adjusted Cox proportional hazards models, gender, age, pathologic lesions (MEST-C), treatment, eGFR and proteinuria were included. Results were expressed as hazard ratios (HR) and 95% confidence intervals (CI). To control the impact of confounding factors among the four groups, we performed propensity-score matching (PSM) according to important clinical and pathologic factors (gender, age, MAP, hypertension, 24-h urine protein, eGFR, treatment, and Oxford MEST-C score). Underweight, overweight and obesity groups were matched to normal weight group with 1:1 nearest neighbor matching without replacement (the caliper width was set at 0.02) to address the marked differences between the groups (9), and the matched cohort was divided into three groups: group1 (normal weight and underweight), group2 (normal weight and overweight) and group3 (normal weight and obesity). Then Kaplan-Meier analyses and Cox proportional hazard models were also conducted to evaluate the risk factors in matched cohort. Besides, the effect modification of obesity and hypertension in IgAN patients was clarified by the synergy index. Statistical significance was considered if p value < 0.05.

## Results

### Demographic and clinicopathological characteristics

Among 1054 patients, 587 patients (55.7%) were normal weight, 75 patients (7.1%) were underweight, 291 patients (27.6%) were overweight and 101 patients (9.6%) were obesity. The mean follow-up time was 61 ± 29 months, the mean age of all patients was 35 ± 11 years, and male patients accounted for 45.2% of the whole cohort. It was noticed that obesity patients had higher rate of unhealthy behaviors, such as smoking (27.7%, p=0.006) and drinking (29.7%, p=0.027). Regarding clinical features, hypertension was observed in 51.5% of patients in obesity group. Furthermore, increased serum creatinine and urine protein levels were associated with higher BMI, especially in obesity patients (p<0.05). In addition, IgAN patients with obesity tended to have higher rates of hypertriglyceridemia, hypercholesterolemia and hyperuricemia (p<0.05). However, no significant difference was found in pathologic lesions (MEST-C) or medical treatments among the four groups (**Table 1**).

**Table 1:** Demographic and clinicopathological characteristics of 1054 unmatched IgAN patients at the time of renal biopsy.

Parameters	Normal weight (N=587)	Underweight (N=75)	Overweight (N=291)	Obesity (N=101)
Male (%)	236 (40.2%)	24 (32%)	160 (55%) ^**^	56 (55.4%) ^**^
Age (years)	31 (26,40)	25 (21,29) ^**^	38 (30,46) ^**^	41 (33,47) ^**^
MAP (mmHg)	95(88,104)	90 (84,99) ^**^	99 (90,107) ^**^	102(92,112) ^**^
Hypertension (%)	121 (20.6%)	9 (12%)	107 (36.8%) ^**^	52 (51.5%) ^**^
BMI (kg/m^2^)	21.2 (19.8,22.7)	17.6 (17,18.3) ^**^	25.5 (24.8,26.6) ^**^	30(28.5,31.7) ^**^
Smoking (%)	96 (16.4%)	4 (5.3%) ^*^	53 (18.2%)	28 (27.7%) ^**^
Drinking (%)	117 (19.9%)	12 (16%)	69 (23.7%)	30 (29.7%) ^*^
Anemia (%)	81 (13.8%)	53 (29.3%) ^**^	30 (10.3%)	7 (6.9%)
24h-proteinuria (g/d)	1.2 (0.7,2.38)	1.0 (0.5,2.16) ^*^	1.47 (0.9,3) ^**^	2.27 (1,3.74) ^**^
Alb (g/L)	40 (36.2,43.1)	40.9 (37.1,44.5)	40.7 (37.1,43.7) ^*^	40.7 (35.7,43.8)
Hypertriglyceridemia (%)	191 (32.5%)	13 (17.3%) ^**^	171 (58.8%) ^**^	72 (71.3%) ^**^
Hypercholesterolemia (%)	209 (35.6%)	13 (17.3%) ^**^	121 (41.6%)	47 (46.5%) ^*^
Hyperuricemia (%)	217 (37%)	16 (21.3%) ^**^	129 (44.3%) ^*^	65 (64.4%) ^**^
eGFR (mL/min/1.73 m^2^)	96 (66,119)	113 (82,130) ^**^	83(61,107) ^**^	81(58,106) ^**^
SCr (umol/L)	80 (64,109)	69(58,96) ^**^	90 (71,114) ^**^	93 (74,119) ^**^
CKD stages				
stage1	327 (55.7%)	54 (72%) ^*^	129 (44.3%) ^**^	44 (43.6%)
stage2	135 (23%)	11 (14.7%)	9 (31.3%)	30 (29.7%)
stage3	110 (18.7%)	7 (9.3%)	65 (22.3%)	24 (23.8%)
stage4	15 (2.6%)	3 (4%)	6 (2.1%)	3 (3%)
Pathology (Oxford classification)				
M1	446 (76%)	53 (70.7%)	203 (69.8%)	75 (74.3%)
E1	21 (3.6%)	5 (6.7%)	12 (4.1%)	7 (6.9%)
S1	360 (61.3%)	39 (52%)	178 (61.2%)	56 (55.4%)
T1/T2	115 (19.6%)	13 (17.3%)	58 (19.9%)	18 (17.8%)
C1/C2	133 (22.7%)	17 (22.7%)	59 (20.3%)	17 (16.8%)
Treatment				
Corticosteroids alone or/and plus immunosuppressant (%)	338 (57.6%)	42 (56%)	175 (60.1%)	63 (62.4%)

MAP, mean arterial pressure; BMI, body mass index; ALB, albumin; eGFR, estimated glomerular filtration rate; SCr, serum creatinine; CKD, chronic kidney disease. M, mesangial proliferation; E, endocapillary proliferation; S, segmental sclerosis; T, tubular atrophy/interstitial fibrosis; C, crescents; IgAN, Immunoglobulin A nephropathy. Other three BMI groups compared with normal weight group, which was used as reference.

*, P value <0.05; **, P value <0.01.

### The relationships between BMI and renal outcomes in IgAN patients

In the unmatched cohort, the percentage of patients progressing to ESKD was 11.30 per 1000 person-years in normal group and 17.68 per 1000 person-years in obesity group. But, there was no significant difference in the results of univariate Cox proportional hazard model and Kaplan-Meier survival analysis (**Table 2**). Then, to investigate the association of BMI with the progression of IgAN by excluding the impact of confounding indicators, we performed PSM to get the matched cohort (**Table 3**). After PSM significant relationships were determined between different groups, compared with normal weight group, patients with underweight presented with higher proportions of mesangial hypercellularity, overweight patients had higher rate of hypertriglyceridaemia. Notably, patients with obesity had worse clinical features, such as higher proteinuria, anaemia and hyperuricaemia. However, BMI did not have a significant influence on the renal outcome of IgAN patients based on the Kaplan-Meier survival and univariate Cox proportional hazard model (**Table 4**).

**Table 2:** The relationships between BMI and renal outcomes in the unmatched cohort.

Subgroup	ESRD (%)	P value of Cox	Hazard Ratio (95%CI)	P value of KM
Group 1		0.819	1.116 (0.436-2.855)	0.819
Normal weight	35 (6%)			
Underweight	5 (6.7%)			
Group2		0.889	1.043 (0.577-1.886)	0.889
Normal weight	35 (6%)			
Overweight	16 (5.5%)			
Group3		0.173	1.714 (0.789-3.723)	0.168
Normal weight	35 (6%)			
obesity	8 (7.9%)			

P value of Cox and KM were analyzed by Univariate Cox proportional hazard model and Kaplan-Meier survival analysis. In each group, normal weight group was used as reference.

**Table 3:** Demographic and clinicopathological characteristics of matched groups.

Parameters	Group1		Group2		Group3	
	Normal weight (N=73)	Underweight (N=73)	Normal weight (N=276)	Overweight (N=276)	Normal weight (N=95)	Obesity (N=95)
Male (%)	18 (24.7%)	24 (32.9%)	149 (54%)	149 (54%)	55 (57.9%)	51 (53.7%)
Age (years)	26 (21,32)	25 (21,29)	37 (29,43)	38 (29,45.8)	38 (32,48)	40 (32,46)
MAP (mmHg)	91.7 (85.3,94.8)	90 (83.5,98.8)	96.7 (90,107.3)	98.7 (90,106.7)	99.7 (92.3,113)	101.7 (91.7,111.7)
Hypertension (%)	3 (4.1%)	9 (12.3%)	87 (31.5%)	93 (33.7%)	44 (46.3%)	46 (48.4%)
BMI (kg/m^2^)	20.7 (19.8,21.8)	17.7 (17,18.3) ^**^	21.6 (20.2,22.7)	25.5 (24.8,26.6) ^**^	21.6 (20.2,23.1)	29.4 (28.5,31.7) ^**^
Smoking (%)	6 (8.2%)	4 (5.5%)	67 (24.3%)	46 (16.7%) ^**^	25 (26.3%)	24 (25.3%)
Drinking (%)	11 (15.1%)	12 (16.4%)	76 (27.5%)	61 (22.1%)	32 (33.7%)	27 (28.4%)
Anemia (%)	13 (17.8%)	21 (28.8%)	41 (14.9%)	29 (10.5%)	17 (17.9%)	7 (7.4%) ^**^
24h-proteinuria (g/d)	0.83 (0.34,1.98)	1 (0.51,2.17)	1.4 (0.7,2.5)	1.5 (0.9,3)	1.7 (0.7,2.7)	2.2 (1,3.7) ^**^
Alb (g/L)	39.8 (37.4,42.8)	40.9 (36.9,44.5)	39.8 (36.3,42.9)	40.7 (37.1,43.5)	39.9 (35,42.6)	40.8 (35.8,43.9)
Hypertriglyceridemia (%)	22 (30.1%)	13 (17.8%)	104 (37.7%)	159 (57.6%) ^**^	40 (42.1%)	68 (71.6%)
Hypercholesterolemia (%)	19 (26%)	13 (17.8%)	104 (37.7%)	117 (42.4%)	41 (43.2%)	44 (46.3%)
Hyperuricemia (%)	19 (26%)	16 (21.9%)	112 (40.6%)	121 (43.8%)	37 (38.9%)	61 (64.2%) ^**^
eGFR (mL/min/1.73 m^2^)	114.5 (81.3,125.8)	112.8 (80.8,129.7)	80.1 (54.8, 109.3)	82.8 (60.7, 107.1)	78.2 (51.6,109.6)	84.9 (62.4,106.4)
SCr (umol/L)	67 (57.7,89.5)	69.1 (58,96.5)	91 (71.8,129.4)	90 (71,114)	94.6 (69.9,136.9)	93 (74,115.9)
CKD stages						
Stage 1	51 (69.9%)	53 (72.6%)	112 (40.6%)	124 (44.9%)	39 (41%)	43 (45.3%)
Stage 2	9 (12.3%)	10 (13.7%)	79 (28.6%)	85 (30.8%)	24 (25.3%)	29 (30.5%)
Stage 3	12 (16.4%)	7 (9.6%)	73 (26.5%)	61 (22.1%)	26 (27.4%)	20 (21.1%)
Stage 4	1 (1.4%)	3 (4.1%)	12 (4.3%)	6 (2.2%)	6 (6.3%)	3 (3.1%)
Pathology (Oxford classification)						
M1	65 (89%)	52 (71.2%) ^**^	196 (71%)	196 (71%)	73 (76.8%)	70 (73.7%)
E1	4 (5.5%)	4 (5.5%)	12 (4.3%)	11 (4%)	5 (5.3%)	4 (4.2%)
S1	34 (46.6%)	39 (53.4%)	157 (56.9%)	108 (60.9%)	46 (48.4%)	53 (55.8%)
T1/T2	10 (13.7%)	13 (17.8%)	63 (22.8%)	54 (19.6%)	20 (21.1%)	18 (18.9%)
C1/C2	20 (27.4%)	17 (23.3%)	62 (22.5%)	57 (20.7%)	15 (15.8%)	16 (16.8%)
Treatment						
Corticosteroids alone or/and plus immunosuppressant (%)	48 (65.8%)	41 (56.2%)	168 (60.9%)	169 (61.2%)	62 (65.3%)	58 (61.1%)

MAP, mean arterial pressure; BMI, body mass index; ALB, albumin; eGFR, estimated glomerular filtration rate; SCr, serum creatinine; CKD, chronic kidney disease. M, mesangial proliferation; E, endocapillary proliferation; S, segmental sclerosis; T, tubular atrophy/interstitial fibrosis; C, crescents. Each group was compared with normal weight, which was used as reference.

*, P value <0.05; **, P value <0.01.

**Table 4:** The relationships between BMI and renal outcomes in the matched cohort.

Subgroup	ESRD (%)	P value of Cox	Hazard Ratio (95%CI)	P value of KM
Group 1		0.252	2.345 (0.545-10.093)	0.240
Normal weight	4 (5.5%)			
Underweight	5 (6.8%)			
Group2		0.729	0.894 (0.473-1.689)	0.729
Normal weight	24 (8.7%)			
Overweight	16 (5.8%)			
Group3		0.415	1.486 (0.573-3.853)	0.413
Normal weight	11 (11.6%)			
obesity	8 (8.4%)			

P value of Cox and KM were analyzed by Univariate Cox proportional hazard model and Kaplan-Meier survival analysis. In each group, normal weight group was used as reference.

### Obesity contributes to risk factors for renal progression and outcomes when combined with hypertension

**Figure 2:**
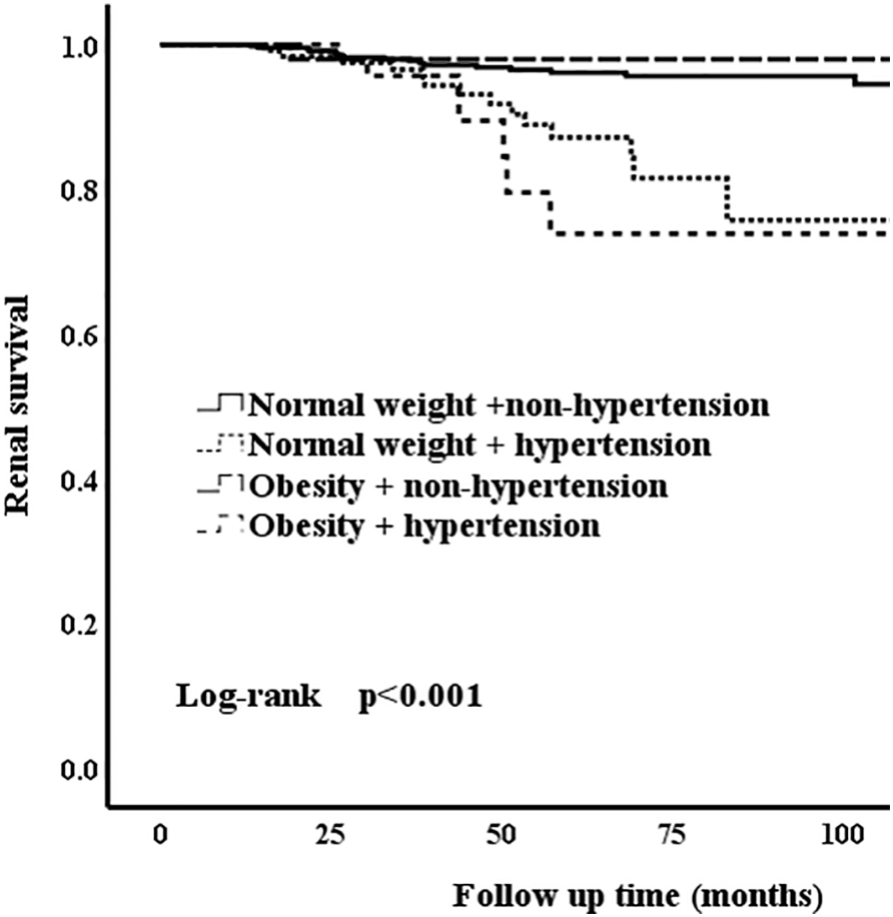
Kaplan-Meier analysis of obesity and hypertension for the unmatched cohort.

**Figure 3:**
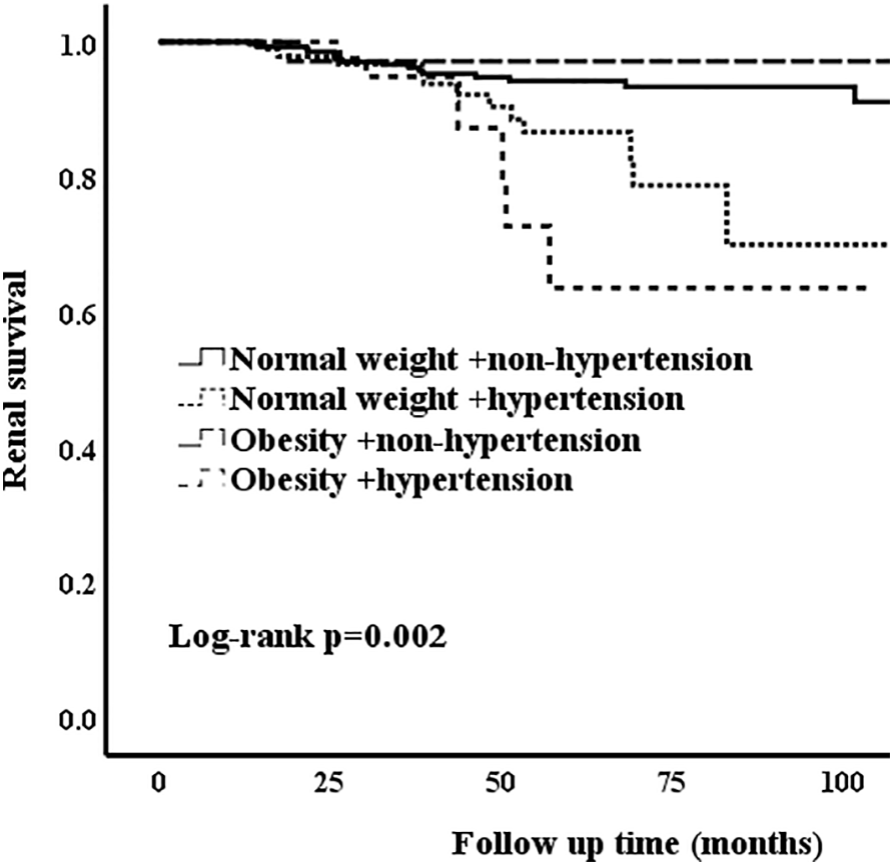
Kaplan-Meier analysis of obesity and hypertension for the unmatched patients with 24-h urine protein ≥ 1 g.

In the unmatched cohort, compared with normal weight patients, obesity groups were associated with higher risk of hypertension. Thus, we conducted a series of analyses to assess whether hypertension and obesity could have an effect on the renal outcomes in IgAN patients. Correspondingly, the patients who presented with normal weight and non-hypertension were considered as the reference group. The Kaplan-Meier analysis revealed that IgAN hypertension patients with obesity had the worse renal outcome (**Figure 2**) **Table 5** shows the results of different multivariate Cox proportional hazards models. Obesity and hypertension increased the risk of ESRD, both adjusted and unadjusted for confounding factors. When unadjusted for confounder factors, patients with obesity and hypertension were associated with a 5.2-fold increased risk of ESRD (p < 0.001). After adjustment for gender, age, pathologic lesions (MEST-C), treatment, eGFR and proteinuria did not affect the trend (HR = 3.246, 95%CI = 1.207-8.726, p = 0.02). Results for the remaining covariates in these multivariate Cox regression analyses are provided in **Supplementary Table 1**. In addition, risk stratification has long been developed as a strategy to predict patient outcomes and potentially alter or optimize comorbidities and modifiable risk. Clinical risk factors, such as persistent proteinuria or hypertension, appear to generally have greater predictive power for the renal outcome (18). Thus, according to the urine protein of the whole unmatched cohort, patients were divided into 24-h urine protein ≥ 1 g or < 1 g per day. We found that hypertension and obesity could accelerate the progression of IgAN, especially for the patients with 24-h urine protein ≥ 1 g (**Figure 3**) In addition, the synergy index was calculated to assess the effect modification of obesity and hypertension on the renal outcome of ESRD, and the synergy index was 1.52, indicating that there was positive interaction between obesity and hypertension in IgAN.

**Table 5:** Univariate and multivariate Cox proportional hazard model for the renal outcome in IgAN patients with obesity and hypertension.

Parameter	Unadjusted		Model 1		Model 2		Model 3	
	HR (95%CI)	P value	HR (95%CI)	P value	HR (95%CI)	P value	HR (95%CI)	P value
Normal weight and non-hypertension	1 (Reference)		1 (Reference)		1 (Reference)		1 (Reference)	
Normal weight and hypertension	4.038 (2.004-8.138)	<0.001	4.307 (2.047-9.060)	<0.001	2.270 (1.057-4.873)	0.035	1.612 (0.755-3.441)	0.217
Obesity and non-hypertension	0.609 (0.081-4.568)	0.630	0.549 (0.072-4.170)	0.562	0.644 (0.084-4.964)	0.673	1.195 (0.149-9.568)	0.867
Obesity and hypertension	5.177 (2.128-12.596)	<0.001	5.154 (1.956-13.579)	0.001	3.767 (1.357-10.458)	0.011	3.246 (1.207-8.726)	0.02

Model 1 was adjusted for gender and age.

Model 2 was adjusted for covariates in model 1 plus Oxford M (mesangial hypercellularity), E (endocapillary proliferation), S (segmental glomerulosclerosis), T (tubular atrophy/interstitial fibrosis), and C (crescent) and Corticosteroids alone or/and plus immunosuppressant (yes or no).

Model 3 was adjusted for covariates in model 2 plus eGFR, 24h-proteinuria≥ 1g (yes or no).

## Discussion

With changes in lifestyle and dietary habits, the prevalence of metabolism-related diseases such as obesity has increased at a rapid rate in developed and developing countries around the world in recent decades (19).

Previously, high BMI was reported as a significant risk factor for progression and prognosis in IgAN (5, 20), and weight reduction was suggested to decrease proteinuria in overweight patients with IgAN to delay renal dysfunction progression (21). Current epidemiologic evidence indicates that obesity is not a risk factor for IgAN (8, 9). However, these studies were confined by the small sample size of the population and no or limited adjustments were conducted for other critical risk factors for IgAN patients. Moreover, the treatment was significantly different between obesity and non-obesity patients. In addition, the influence of obesity on IgAN patients may be confused by some confounding factors. Therefore, the association between obesity and IgAN still remains unclear.

Consistent with some previous studies, the main finding of this retrospective study of 1054 patients with IgAN is that being obesity at the time of renal biopsy significantly correlated with more severe clinical features and increased proteinuria and favored the subsequent development of ESRD. In our current study, all the patients were divided into four groups: underweight, normal weight, overweight and obesity. IgAN patients with obesity tended to be old, male and have severe proteinuria and renal dysfunction. Besides, they always had poor healthy lifestyle, smoking and alcohol drinking. More importantly, in our study, obesity patients were noted to have metabolic syndrome, including but not limited to hypertension, hyperuricemia, hypertriglyceridemia and hypercholesterolemia. It was reported that high BMI indirectly accelerated the progression of IgAN by inducing metabolic syndrome. Additionally, hypertension, hypertriglyceridemia and hyperuricemia, which contribute to metabolic syndrome, are strongly related to the development of ESRD (22). As a result, it is worth assessing and paying attention to the risk of obesity.

Obesity can induce afferent arteriole vasodilation and high intraglomerular pressures in order to augment glomerular filtration rate to meet the higher metabolic demands. As an individual gains weight, the glomerular and glomerular capillary diameter might increase, leading to terminally differentiated podocytes covering a larger glomerular capillary surface and glomerular capillary wall tension increasing. These were associated with an increased risk of proteinuria (23).

There was no significant difference among the four groups regarding the pathological lesions of MEST-C and treatment after renal biopsy. Because obesity-related glomerulopathy (ORG) is characterized by glomerulomegaly in the presence or absence of focal and segmental glomerulosclerosis lesions (24), more obesity IgAN patients are needed to investigate pathologic lesions.

A previous study revealed that hypertension and proteinuria increased the risk of ESRD in IgAN patients (25). However, our results showed that obesity was not a direct and independent risk factor for IgAN, and the combination of obesity and hypertension might be a risk indicator for IgAN. In clinical practice, many patients are exposed to the combined effects of obesity and hypertension. Our study found that such exposure is associated with a higher risk for ESRD. Furthermore, the positive interaction between obesity and hypertension in IgAN patients was verified by our results.

These results indicated that obesity may not be an independent risk factor for poor renal outcome, but to some extent, coexists with hypertension could increase the risk of ESRD in IgAN patients. When obesity coexists with common risk factors, such as hypertension, it may further aggravate the patient’s disease progression. Based on our results, we suggest that IgAN patients, especially those with hypertension, should strictly control their weight and prevent obesity, so as to reduce the risk of poor renal prognosis.

The study had three main limitations. First, the body mass index was calculated at the time of renal biopsy and the groups were classified by those data. which may lead to different categorization over time. Besides, it was a retrospective study and only in a single hospital center. As a result, Multi-center studies would be useful to verify the findings of this study. In addition, the mean follow-up time of 61 months was relatively short, especially for IgAN, which is a slowly progressing disease. Though we tried our best to collect all the information retrospectively, a total of 192 patients (12%) with missing data were excluded from the study, which may have affected renal outcome. However, because of their small number, we do not think it would have had a significant impact on our findings. In addition, we are expanding the cohort population and extending the follow-up time, and expect to find more compelling results in the future.

## Conclusions

Obesity had an effect on the progression of IgAN when combined with hypertension. In addition, hypertension was common in obesity-IgAN patients.

## Data availability statement

The raw data supporting the conclusions of this article will be made available by the authors, without undue reservation.

## Ethics statement

Written informed consent was obtained from the individual(s) for the publication of any potentially identifiable images or data included in this article.

## Author contributions

Research idea and study design: WQ and SW. Data acquisition: WQ, SW, and LD.Data analysis and interpretation: WQ, SW and JT. Statistical analysis: SW, XZ andAQ. Supervision: WQ. Each author accepted accountability for the overall work by ensuring that questions pertaining to the accuracy or integrity of any portion of the work are appropriately investigated and resolved. All authors contributed to the article and approved the submitted version.
