# Supplementary material for: Association of obesity with the development of end stage renal disease in IgA nephropathy patients
Source: Front Endocrinol (Lausanne). 2023 Mar 20;14:1094534. doi: 10.3389/fendo.2023.1094534 (PMC10067555; doi:10.3389/fendo.2023.1094534)
Supplement: Supplementary file 1 [file Table_1.docx]

Supplementary Table 1. The results for the remaining covariates in the multivariable Cox regression analyses presented in Table 5.

|  | HR | 95%CI | P |
| --- | --- | --- | --- |
| **Model1** |  |  |  |
| Gender | 2.472 | 1.298-4.704 | 0.006 |
| Age | 0.982 | 0.952-1.013 | 0.257 |
| **Model2** |  |  |  |
| Gender | 1.580 | 0.796-3.134 | 0.191 |
| Age | 0.980 | 0.945-1.016 | 0.267 |
| M | 3.161 | 0.738-13.546 | 0.121 |
| E | 0.844 | 0.230-3.104 | 0.799 |
| S | 0.801 | 0.395-1.625 | 0.539 |
| T | 8.384 | 3.985-17.640 | <0.001 |
| C | 1.330 | 0.643-2.750 | 0.442 |
| Treatment | 0.788 | 0.405-1.535 | 0.485 |
| **Model3** |  |  |  |
| Gender | 1.213 | 0.603-2.442 | 0.588 |
| Age | 0.945 | 0.907-0.985 | 0.007 |
| M | 2.376 | 0.549-10.276 | 0.247 |
| E | 0.659 | 0.171-2.540 | 0.545 |
| S | 0.858 | 0.408-1.802 | 0.685 |
| T | 2.760 | 1.229-6.201 | 0.014 |
| C | 0.837 | 0.950-0.979 | 0.637 |
| Treatment | 0.617 | 0.308-1.236 | 0.173 |
| eGFR | 0.965 | 0.950-0.979 | <0.001 |
| 24h-proteinuria | 3.526 | 1.194-10.412 | 0.023 |

Abbreviations: eGFR, estimated glomerular filtration rate; M, mesangial proliferation; E, endocapillary proliferation; S, segmental sclerosis; T, tubular atrophy/interstitial fibrosis; C, crescents.
